# Effect of Cyclic Ice Plug Deformation on Microstructure and Mechanical Behaviors of Nuclear-Grade Low-Carbon Tubular Steel

**DOI:** 10.3390/ma17112642

**Published:** 2024-05-30

**Authors:** Minglei Hu, Wei Zhang, Ke Xu, Bin Hu, Dongsheng Li, Lan Wang, Rencai Liu, Xiaohua Zhao

**Affiliations:** 1 China Nuclear Power Operation Management Co., Ltd., Haiyan 314300, China; 2 School of Materials Science and Engineering, Jiangsu University, Zhenjiang 212013, China

**Keywords:** nuclear-grade 20# pipeline steel, cyclic freeze–thaw ice plugging, plastic deformation, dislocation morphology, mechanical properties

## Abstract

This study subjected nuclear-grade 20# pipeline steel to cyclic freeze–thaw ice plugging tests, simulating the plastic deformation experienced by pipes during ice plug removal procedures. Subsequently, the dislocation morphology and mechanical properties of the specimens post cyclic ice plugging were examined. The cyclic ice plugging process led to an increase in the dislocation density within the specimens. After 20 and 40 cycles of ice plugging, the internal dislocation structures evolved from individual dislocation lines and dislocation tangles to high-density dislocation walls and dislocation cells. These high-density dislocation walls and cells hindered dislocation motion, giving rise to strain hardening phenomena, thereby resulting in increased strength and hardness of the specimens with an increasing number of ice plugging cycles. In addition, a large stress field was generated around the dislocation buildup, which reduced the pipe material’s plastic toughness. The findings elucidate the effects of cyclic ice plugging on the microstructure and properties of nuclear-grade 20# pipeline steel, aiming to provide a theoretical basis for the safe and stable application of ice plugging technology in nuclear piping systems.

## 1. Introduction

Compared to fossil fuels like oil and gas, nuclear power generation is recognized as a cleaner, low-carbon, and efficient baseload energy source [1]. Nuclear power is suitable for providing both baseline capacity and essential peak-shaving services in large-scale power grids, making it a practical choice for global energy strategies in major nuclear nations and witnessing increasing development worldwide. Moreover, nuclear energy plays a proactive role in addressing global climate change, and its robust development aligns with the trend in world energy utilization, holding significant importance [2,3,4]. Nonetheless, while nuclear power effectively contributes to the transition toward cleaner and lower-carbon energy, it inherently carries a higher degree of risk [5]. Hence, ensuring the safe operation of nuclear power systems becomes critically important, as the safety of nuclear power largely depends on the safe functioning of its equipment. Consequently, regular inspection and maintenance of the pipeline systems in nuclear power plants are necessary. Within numerous liquid-based pipeline systems in nuclear power plants, when devices requiring maintenance, replacement, or technical upgrades cannot be isolated individually due to operational constraints, a specialized online isolation measure must be adopted to guarantee the safety and stable operation of the units. Ice plug-freezing isolation technology, abbreviated as ice plugging, represents an economically viable solution that effectively addresses this challenge by serving as a system isolation technique [6].

Ice plugging technology involves the localized cooling of a system pipeline requiring isolation using refrigerants, such as liquid nitrogen or dry ice, causing the liquid medium within that segment to freeze, forming a stable ice plug within the pipe, thereby achieving fluid isolation [7,8]. During the replacement of pressure piping in a heavy-water reactor (HWR) nuclear power plant, it is necessary to establish isolation between the pressure piping and the main heat transfer system [9]. However, due to equipment construction, there is no isolation valve present between the pressure piping and the main heat transfer piping. Consequently, traditional isolation methods would require additional investment in materials and equipment and potentially even partial equipment shutdown, resulting in significant economic losses. In contrast, ice plug technology offers an effective solution by isolating the piping without the need to shut down or discharge the entire system, thereby reducing the risk of radioactive material leakage [8,9]. This technique boasts simplicity, safety, and economic benefits, further enhancing the maintenance capabilities of nuclear power plants [6,8,9]. However, the formation of an ice plug within a pipe involves large temperature gradients and rapid local freezing processes. Due to the complex interactions among water molecules within the liquid medium, water expands when it freezes, causing radial expansion and deformation between the pipe wall and the ice. This results in substantial stresses within the pipe [9,10]. It is well-known that stress accelerates intergranular corrosion and intergranular stress corrosion cracking in steel, introducing failure risks to the piping process system [11,12,13,14,15]. Research by Le Li et al. [11] revealed that, under the corrosive environments encountered by low-carbon steel pipes, the stress applied to the steel expedites corrosion processes, leading to a decline in the material’s mechanical properties, particularly its ultimate strength and fracture strain. Given the need to maintain the safe and stable operation of nuclear equipment, ice plugging may be carried out multiple times at the same location within a process system. Repeated cycles of ice plugging can result in cumulative minor deformations of the pipe wall, preventing the pipe from self-recovering through its plastic properties. This, in turn, affects the service performance of the pipe wall, potentially causing pipe rupture and posing safety hazards to the nuclear equipment.

During cyclic plastic deformation, the microstructure of materials undergoes changes, notably in the distribution of dislocations [16,17,18,19,20,21]. Dislocation arrangement is determined by dislocation motion and their mutual interactions, primarily involving dislocation multiplication and rearrangement. Common arrangements of dislocations in plastically deformed metals include dislocation walls, dislocation cells, and even subgrain boundaries [17,22,23,24]. It is widely acknowledged that microstructural variations affect the mechanical behavior of materials [16,24,25,26,27,28,29,30,31]. Y.Z. Li et al. [30] devised an optimized double-reduction process that leverages control over dislocation density and bake-hardening effects. This process enhances the uniform elongation of ultra-thin steel plates significantly without compromising yield stress, thereby offering an effective approach to improve the formability of high-yield-stress ultra-thin steel plates. Through transmission electron microscopy (TEM), changes in dislocations under cyclic stress conditions can be observed [16,17,28], revealing that a uniform distribution of dislocations can improve a material’s long-term service performance [24,27,29]. Conversely, the accumulation of dislocations can initiate fatigue cracks, raising safety concerns during the use of steel materials [16,25]. Therefore, investigating the effects of freezing and thawing cycles on nuclear-grade low-carbon steel pipes, specifically the thermal expansion and contraction caused by the solidification and melting of the internal liquid medium, becomes highly relevant. These processes can shed light on the implications for material integrity and performance after repeated ice plugging operations.

This study analyzes nuclear-grade low-carbon pipeline steel samples in their original state and after undergoing various numbers of ice plugging cycles to investigate their evolving mechanical behavior and dislocation morphologies. Concurrently, it explores the relationship between the changes in dislocation patterns due to plastic deformation and the associated mechanical properties. The aim is to provide a theoretical foundation for the safe application of ice plugging technology in the maintenance of nuclear power plant process systems.

## 2. Materials and Methods

### 2.1. Experimental Materials

The pipes used in the circulating water system of the studied nuclear power plant were provided by Qinshan Nuclear Power Co., Ltd. (Qinshan, China). These pipes conform to the specifications outlined in GB/T 3087-2022 [32] for ‘Carbon Seamless Steel Tubes for Low and Medium Pressure Boilers’, utilizing Grade 20 steel. The chemical composition of the steel was determined using a direct-reading spectrometer (SPECTRO MAXx mm06, SPECTRO, Kleve, Germany), and the results are presented in Table 1. The dimensions of the pipes are Φ49 × 6 mm.

### 2.2. Ice Plugging Freeze Isolation Test (Ice Plug Technology)

**Figure 1:**
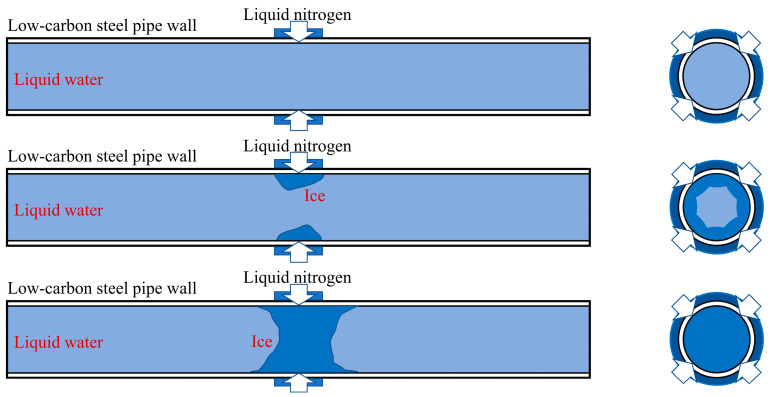
Schematic diagram of the ice plugging process.

Figure 1 presents a schematic diagram of the ice plugging process. Here, an outer jacket device is fixed at a chosen position on the pipe’s outer surface, serving as a reservoir for storing the refrigerant (liquid nitrogen) during the ice plugging procedure. When liquid nitrogen is introduced into the annulus surrounding the pipe, it exchanges heat with the liquid medium (water) inside the pipe, causing the water to cool and freeze from the pipe wall radially inward, eventually forming an ice plug capable of withstanding a certain pressure to block the pipe and isolate upstream and downstream maintenance equipment [6,9]. The formation of the ice plug primarily involves two stages: firstly, the heat transfer process between the media on either side of the pipe wall; secondly, the phase transition process wherein the water inside the pipe wall freezes when its temperature drops below 0 °C. During the ice plug test, the stress variation in the pipe wall is monitored using a cryogenic strain gauge and a stress–strain tester. Simultaneously, the temperature fluctuation in the pipe wall is measured by employing a thermocouple. The thermocouple has a measuring range of −200 °C to 200 °C, whereas the cryogenic strain gauge operates within the temperature range of −196 °C to 120 °C. To ensure that the acquired strain data remain unaffected by the cold shrinkage of the strain gauge, the temperature error is rectified using the bridge compensation method at the same temperature.

For this experiment, a repetitive ice plugging cycle approach was adopted, where the formation of an ice plug until its elimination constituted one complete ice plugging cycle test. To simulate actual maintenance scenarios more closely, after the ice plug formed, the supply of liquid nitrogen was maintained for an additional 10 min before ceasing the refrigerant supply. Subsequently, the jacket device was removed, and the ice plug was allowed to melt naturally at room temperature. For pipe specimens subjected to 0, 3, 20, and 40 cycles of ice plugging, samples were obtained at the midway point of the pipe wall where the ice plug was formed. The outer diameter of the pipe within the same cross-section was measured multiple times, and three valid measurements were taken using vernier calipers. These measurements were utilized to calculate the uncircularity factor (*r*).

### 2.3. Mechanical Properties’ Testing

**Figure 2:**
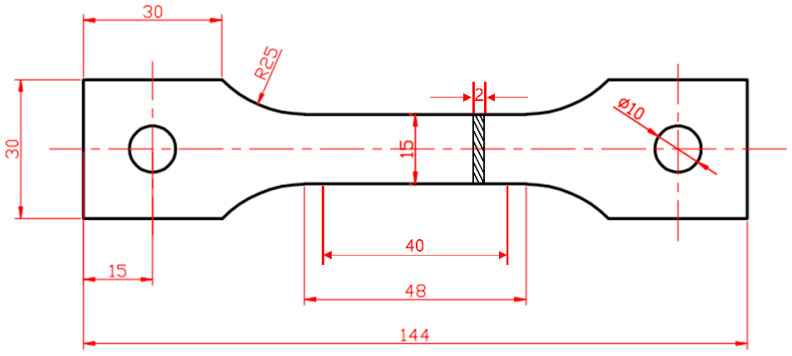
Dimensions of tensile specimen.

Tensile tests were performed at room temperature (25 °C) using a DDL100 electronic universal testing machine. The specimens for tensile testing were extracted from the pipe in the axial direction, as illustrated in Figure 2, with dimensions given in millimeters. The loading conditions were regulated by maintaining a constant displacement rate of 1 × 10^−3^ mm/s. Each experimental condition was replicated three times, with three specimens tested accordingly.

**Figure 3:**
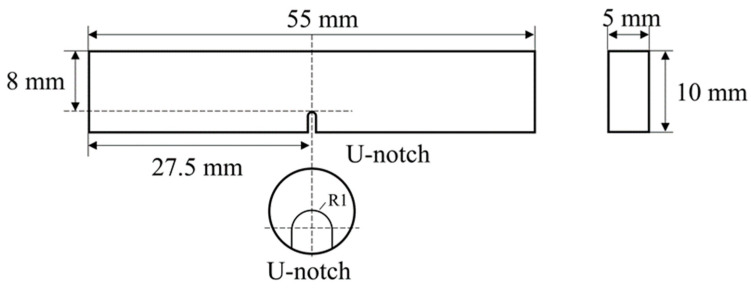
Schematic illustration of the Charpy impact specimen.

Due to the constraint imposed by the thickness of the pipe wall, standard impact specimens could not be prepared. Therefore, referring to GB/T 229-2020 [33] ‘Metallic Materials-Charpy Impact Test Method’, U-notch Charpy impact specimens with dimensions of 55 mm × 10 mm × 5 mm were cut, featuring a U-notch depth of 2 mm, as illustrated in Figure 3. Under each testing condition, three specimens were tested on a pendulum impact tester (NI 300), and the average value of each set was recorded.

Microhardness tests were conducted on different specimens using an automated microhardness tester (FM-ARS 900 (Future-Tech Corporation, Kawasaki, Japan) with a load of 100 gf, a testing interval of 50 μm, and a dwell time of 15 s). The microhardness was measured in Vickers’ hardness units (HV), with measurements taken radially from the inner wall towards the outer wall of the pipe.

### 2.4. Microstructural Observations

Selected specimens in their original state and after various cycles of ice plugging were subjected to microstructural examination. For clarity, the specimens were labeled as follows: the original sample was denoted as sample 0#; the sample after 3 cycles of ice plugging was designated as sample 1#; the sample following 20 cycles of ice plugging was marked as sample 2#; and the specimen that underwent 40 cycles of ice plugging was named sample 3#. Field emission scanning electron microscopy (SEM: FEI NovaNano450, FEI Company, Hillsboro, OR, USA) was employed to observe the fracture surfaces of the impact-tested specimens. Additionally, transmission electron microscopy (TEM) was utilized to characterize the microstructures of the specimens under different testing conditions. TEM samples were prepared using electro-polishing with a double-jet technique, employing a mixture solution composed of 10% perchloric acid and 90% absolute ethanol. The dislocation densities in different samples were determined using an X-ray diffractometer (XRD: Rigaku SmartLab, Rigaku Corporation, Tokyo, Japan, equipped with a Cu target, operating at a voltage of 45 kV and a current of 200 mA).

## 3. Results and Discussion

### 3.1. Stress and Plastic Changes in Pipe Walls

**Figure 4:**
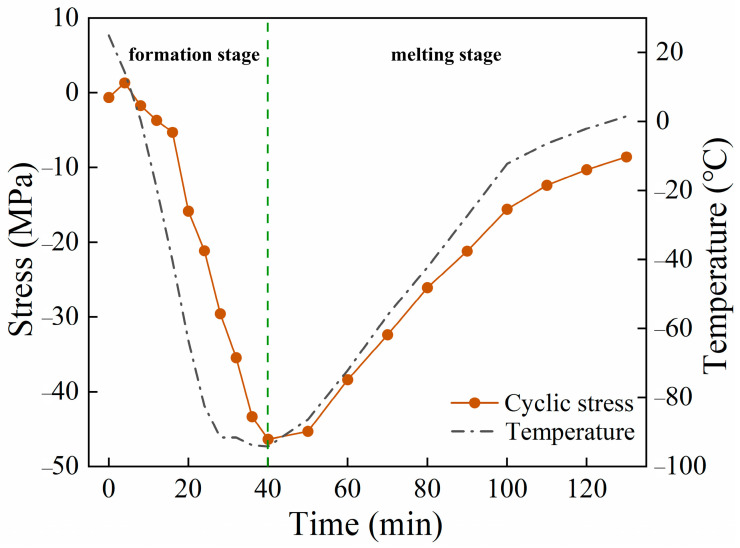
Curves of pipe wall stress and temperature versus time at the location of ice plug formation.

Figure 4 illustrates the curves representing the temporal variations in cyclic stress and temperature of the tube wall at the location where the ice plug forms during the ice plug test. The plot demonstrates that, during the ice plug formation stage, as liquid nitrogen is continuously supplied, the pipe wall temperature decreases further. In contrast, the cyclic stress on the pipe wall continues to increase. Upon reaching the 40 min mark, when the liquid nitrogen supply ceases, the pipe wall cyclic stress reaches its minimum value of −46 MPa. Subsequently, during the ice plug melting stage, the cyclic stress on the pipe wall gradually diminishes until the completion of the test. These observed alterations in cyclic stress on the pipe wall indicate an increase in compressive stress on the pipe during the ice plug test, potentially influencing the pipe material.

Following the conclusion of the ice plug cycling test, the uncircularity factor (*r*) was computed for the tube section wall of the ice plug, maintaining consistent cross-sectional dimensions. The formula employed for this computation is as follows:

$$r=\frac{2(D_{max}-D_{min})}{D_{max}+D_{min}}\times 100\%$$

The variables $D_{max}$ and $D_{min}$ represent the measured maximum and minimum outer diameters of the same pipe cross-section, respectively. After sampling, testing, and calculations, the uncircularity factors (*r*) for specimens 0#, 1#, 2#, and 3# were determined as 0.11%, 0.08%, 0.33%, and 0.42%, respectively. These values fall within the allowable error range specified in GB/T 17395-2008 [34]. Furthermore, it is observed that the uncircularity factor tends to increase with the number of ice plug cycles, indicating a slight degree of plastic deformation induced by the cycling process. Nevertheless, these deformations remain within acceptable limits and meet the service requirements.

### 3.2. Mechanical Properties after Ice Plug Cycling

**Figure 5:**
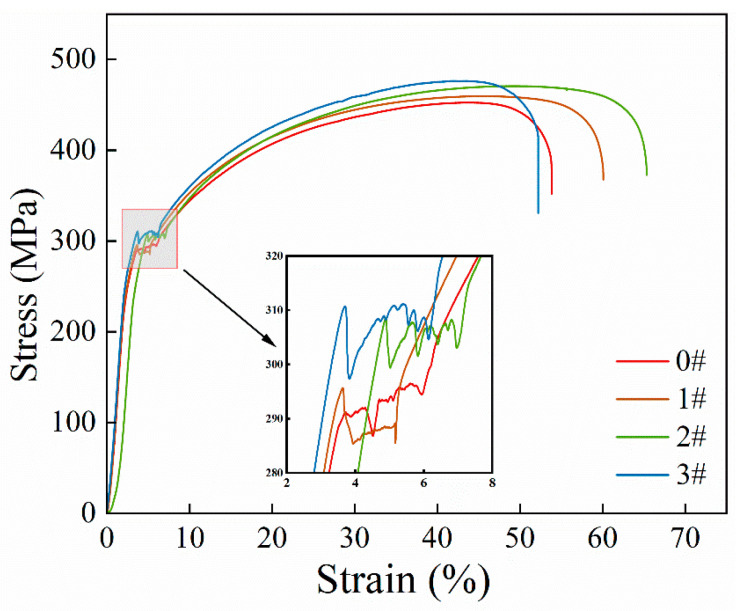
Stress–strain curves for specimens 0#, 1#, 2#, and 3#.

The tensile properties of samples 0#, 1#, 2#, and 3# are depicted in Figure 5. It is evident that both the yield strength and tensile strength increase with the number of ice plugging cycles while the elongation decreases. Table 2 provides the specific values for each sample’s yield strength, tensile strength, and total elongation. Sample 0# has a yield strength of 285.56 MPa, a tensile strength of 452.71 MPa, and a total elongation of 42.35%. For sample 1#, the respective values are 286.12 MPa, 459.77 MPa, and 42.01%. Sample 2# exhibits a yield strength of 299.38 MPa, a tensile strength of 470.92 MPa, and a total elongation of 40.19%. Lastly, sample 3# demonstrates a yield strength of 301.15 MPa, a tensile strength of 477.48 MPa, and a total elongation of 39.20%.

**Figure 6:**
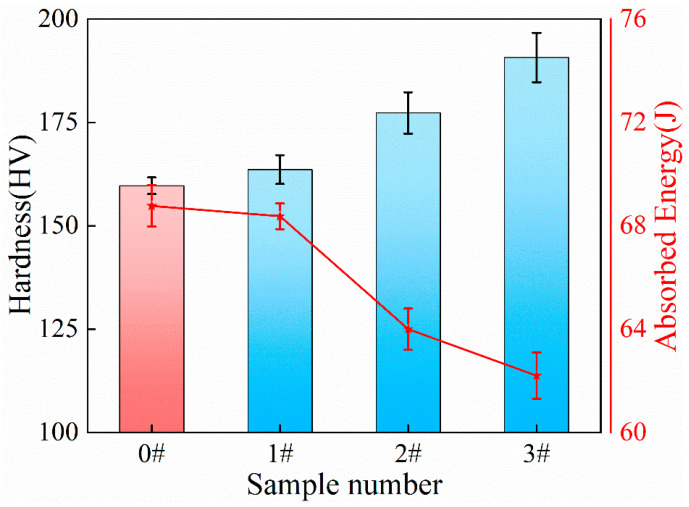
Variation in microhardness and impact absorption energy for specimens subjected to different numbers of ice plugging cycles.

Figure 6 illustrates the variation in the relationship between the mean values of hardness and impact-absorbed energy for the specimens under different ice plugging cycle conditions. It can be seen from Figure 6 that the Vickers’ hardness values for samples 0#, 1#, 2#, and 3# are 159.68 HV, 163.61 HV, 177.29 HV, and 190.71 HV, respectively, while the corresponding mean impact-absorbed energies are 68.77 J, 68.37 J, 64 J, and 62.2 J. From Figure 6, it can also be discerned that the hardness of the specimens increases as the number of ice plugging cycles rises. Contrarily, the impact-absorbed energy of the specimens decreases with an increase in the number of ice plugging cycles. This trend aligns with the general rule governing the change in plastic toughness due to strain hardening in low-carbon steel [11,35].

**Figure 7:**
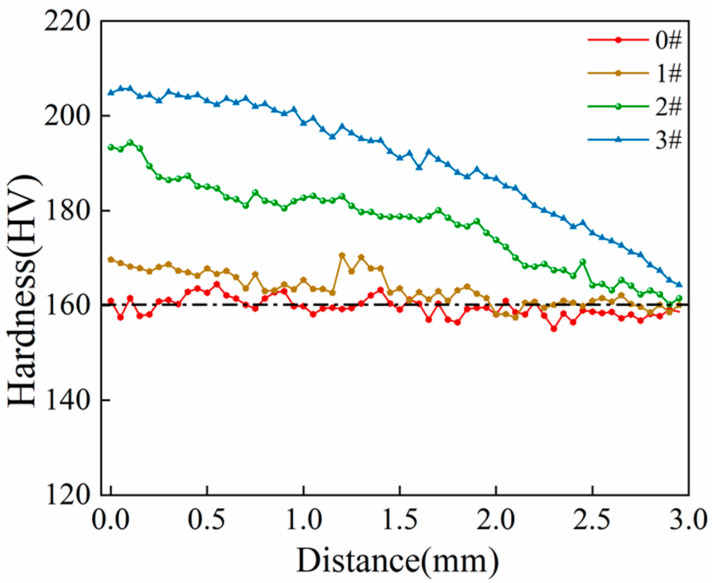
The trend in microhardness variation with respect to the distance from the pipe inner wall for specimens subjected to different ice plugging cycles.

Figure 7 depicts the variation trend in Vickers’ hardness across the distance from the inner pipe wall for the different specimens. From Figure 7, it can be observed that the hardness near the inner pipe wall is higher for specimens that have undergone ice plugging cycles. This phenomenon is attributed to the fact that the volume expansion of the liquid medium during ice plug formation exerts a more pronounced compressive effect on the inner pipe wall compared to the outer wall. The hardness at the inner pipe wall of sample 3# distinctly increases relative to the other specimens, suggesting that strain hardening is more evident under these experimental conditions.

**Figure 8:**
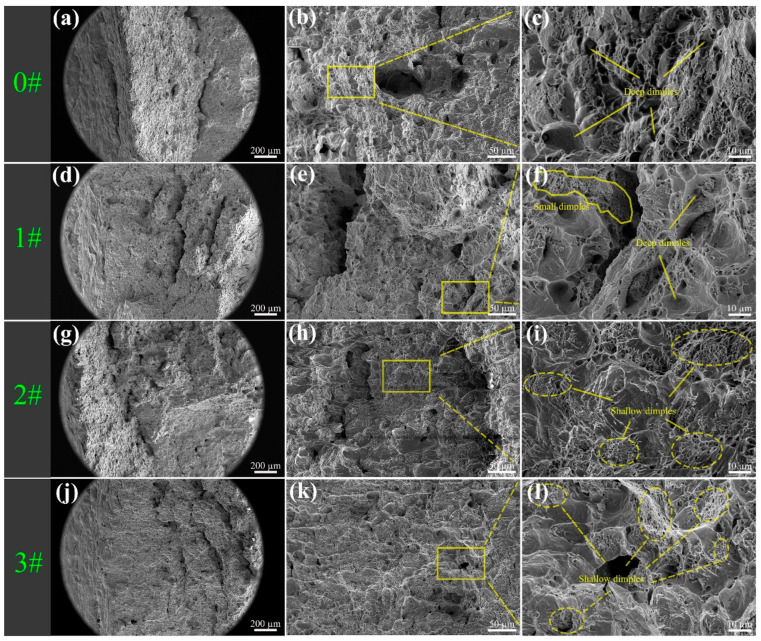
SEM images of fracture surfaces of specimens subjected to various ice plugging cycles: (**a**–**c**) fracture morphology of specimen 0#, (**a**) is the macroscopic morphology of the fracture, (**c**) is an enlargement of the yellow boxed area in (**b**); (**d**–**f**) fracture morphology of specimen 1#, (**d**) is the macroscopic morphology of the fracture, (**f**) is an enlargement of the yellow boxed area in (**e**); (**g**–**i**) fracture morphology of specimen 2#, (**g**) is the macroscopic morphology of the fracture, (**i**) is an enlargement of the yellow boxed area in (**h**); (**j**–**l**) fracture morphology of specimen 3#, (**j**) is the macroscopic morphology of the fracture, and (**l**) is an enlargement of the yellow boxed area in (**k**).

To further investigate the influence of varying ice plugging cycles on the ductility of the pipe specimens, the fracture surfaces were examined under SEM. The micro-morphology of the fracture surfaces is depicted in Figure 8. In Figure 8a,d,g,j, it can be clearly observed that all the specimens exhibit noticeable necking characteristics, indicative of a ductile fracture mode. However, upon closer scrutiny of the remaining fracture microstructures, it is evident that samples 0# and 1# present abundant deep and wide dimples, whereas the fracture surfaces of samples 2# and 3# are relatively flat, characterized by a multitude of smaller dimples and micropores. The microscopic features of the fracture surfaces align consistently with the trend in ductility changes as reflected by the impact absorption energy measurements.

### 3.3. Dislocation Characterization after Ice Plug Cycling

**Figure 9:**
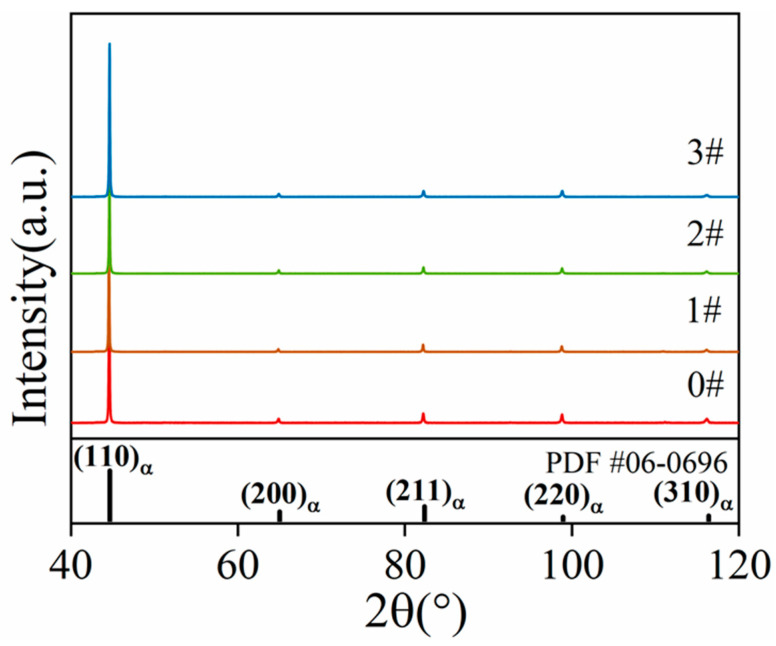
XRD patterns of specimens subjected to different ice plugging cycles.

The X-ray diffraction (XRD) patterns are presented in Figure 9. The diffraction peaks for each specimen’s microstructure consist of five Bragg reflections characteristic of the body-centered cubic (BCC) structure: (110), (200), (211), (220), and (310). Following the ice plugging cycles, no new diffraction peaks appeared for samples 1#, 2#, and 3#, indicating that no new precipitated phases were formed. The dislocation density (*ρ*) was calculated for each specimen after different numbers of ice plugging cycles using the modified Williamson–Hall (MWH) method. The expression for the dislocation density derived from the modified Williamson–Hall equation is as follows [36]:

$$∆K≅0.9/D+{\left(\pi M^{2}b^{2}/2\right)}^{1/2}\rho^{1/2}\left(K{\bar{C}}^{1/2}\right)+O(K^{2}\bar{C})$$

In the formula, *K* = 2*sinθ*/*λ* represents the inverse of the lattice spacing, Δ*K* = 2*cosθ*(Δ*θ*)/*λ* denotes the width of the corresponding peak relative to *K*, where θ signifies the Bragg diffraction angle, and Δ*θ* indicates the width of the corresponding peak relative to θ. The parameter λ is the wavelength of the X-ray ($\lambda_{Cu}$ = 1.5406 $\dot{A}$ = 0.15406 nm). *D* represents the average grain size, b = 0.248 nm is the magnitude of the Burgers vector [37], and ρ denotes the dislocation density. A value of *M* > 1 suggests a random dislocation arrangement, while *M* < 1 implies a highly correlated arrangement. In the present study, the value of *M* is taken to be 1, as provided in Sallez et al. (2015) [38].

The average dislocation contrast factor is represented by $\bar{C}$ [30];

$$\bar{C}={\bar{C}}_{h_{00}}(1-q\frac{h^{2}k^{2}+k^{2}l^{2}+h^{2}l^{2}}{h^{2}+k^{2}+l^{2}})$$

where *h*, *k*, and *l* correspond to the crystallographic index (hkl). ${\bar{C}}_{h_{00}}$ and *q* are two constants that depend on the elastic constants C11, C12, and C44 of the material (Kim and Johnson, 2007) [39], and their relationship is described by Ungár et al. (1999) [40]. Taking the room temperature elastic constants for ferrite as C_11_ = 273 GPa, C_12_ = 110 GPa, and C_44_ = 82 GPa, ${\bar{C}}_{h_{00}}$ is, thus, determined to be 0.160 and q 0.605. Consequently, the dislocation density (*ρ*) is obtained from the slope of the curve relating Δ*K* to $K{\bar{C}}^{1/2}$:

$$\rho =\frac{2\beta^{2}}{\pi M^{2}b^{2}}$$

Here, *β* represents the slope of the curve between Δ*K* (nm^−1^) and $K{\bar{C}}^{1/2}$ (nm^−1^), which is used to calculate the dislocation density. The value of *β* is determined through a linear fit between ΔK and $K{\bar{C}}^{1/2}$ obtained by the least-squares method.

**Figure 10:**
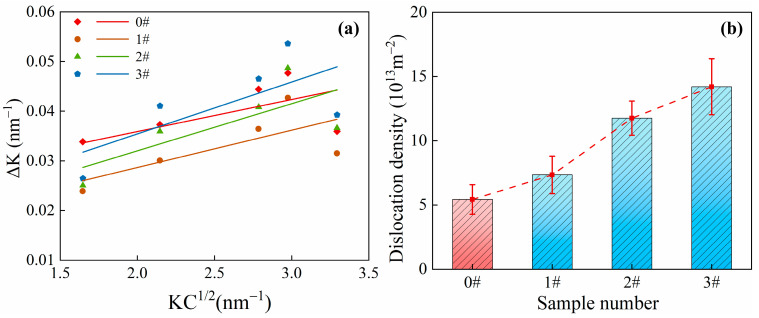
(**a**) Revised Williamson–Hall diagram of each sample under different ice plug cycle conditions; and (**b**) dislocation density maps of samples under different ice plug cycles.

Through the analysis of the XRD patterns for each specimen depicted in Figure 9 and the subsequent numerical calculations, the values of Δ*K* and $K{\bar{C}}^{1/2}$ corresponding to each diffraction peak can be obtained. These data are used to determine the slope in the Williamson–Hall equation. The linear fitting relationship between Δ*K* and $K{\bar{C}}^{1/2}$ for pipe specimens under different ice plugging test conditions is displayed in Figure 10a. As shown in Figure 10a, the slope *β* of the linear relationship corresponding to the red-marked sample 0# is less than the slopes *β* of samples 1#, 2#, and 3#. According to Equation (2), since *ρ* is directly proportional to *β*, this observation suggests that the dislocation densities of samples 1#, 2#, and 3# are higher than that of sample 0#. Figure 10b presents the dislocation densities calculated for each specimen using the aforementioned formula. From Figure 10b, it can be discerned that the dislocation densities of samples 0#, 1#, 2#, and 3# are 5.43 × 10^13^ m^−2^, 7.34 × 10^13^ m^−2^, 1.18 × 10^14^ m^−2^, and 1.42 × 10^14^ m^−2^, respectively. This indicates that the dislocation density in the specimens increases with an increase in the number of ice plugging cycles. Consequently, a higher dislocation density leads to strain hardening, which, in turn, results in elevated microhardness and decreased toughness in the specimens.

**Figure 11:**
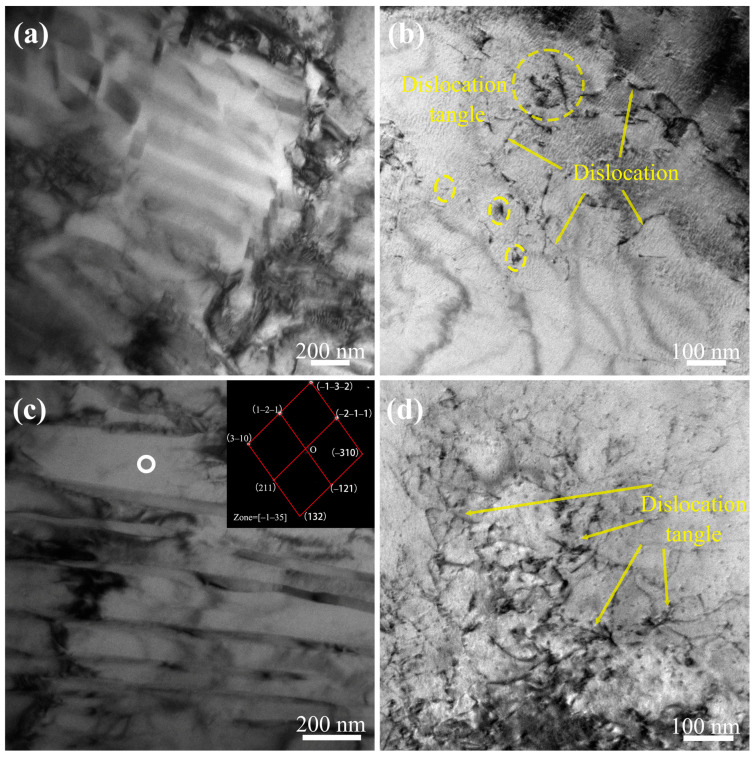
TEM micrographs of samples 0# (**a**,**b**) and 1# (**c**,**d**) and the corresponding SAED patterns of white-circle precipitated phases (**c**).

To deeply understand strain hardening and the deformation behavior in pipe specimens following cyclic ice plugging, the TEM test was employed to examine the microstructural evolution of specimens subjected to different numbers of ice plugging cycles. Figure 11 presents TEM micrographs and selected area electron diffraction patterns for the original specimen (sample 0#) and the specimen after three cycles of ice plugging (sample 1#). In Figure 11a,b, it can be discerned that sample 0# exhibits a microstructure with the sporadic distribution of dislocation points and occasional dislocation tangles, which is consistent with previous research findings [17,18]. The bright-field image and the corresponding selected area electron diffraction pattern (inset) of sample 1# are shown in Figure 11c. It is evident from Figure 11c that, compared to sample 0#, sample 1# experiences a significantly increased dislocation density after three cycles of ice plugging. Moreover, some dislocations have undergone a slip and have interacted with others, resulting in the formation of a small number of dislocation tangles. This is attributed to sample 1# accommodating the thermally induced strain caused by the ice plug’s compressive action through dislocation glide. The selected area electron diffraction pattern (inserted in Figure 11c) confirms that sample 1# consists of a body-centered cubic ferrite phase, which is in line with XRD characterizations. Figure 11d reveals that a large number of dislocations have formed within the specimen, predominantly in the form of tangled dislocations. As in previous studies, the evolution of dislocations is a dynamic competitive process, characterized by dislocation multiplication, the formation of tangled dislocations, the reversible backsliding of dislocations, and the annihilation or dissolution of dislocation tangles [19,22,24,30]. Consequently, as deformation progresses, dislocation tangles accumulate, leading to the development of a large number of low-density tangled dislocations, as depicted in Figure 11d.

**Figure 12:**
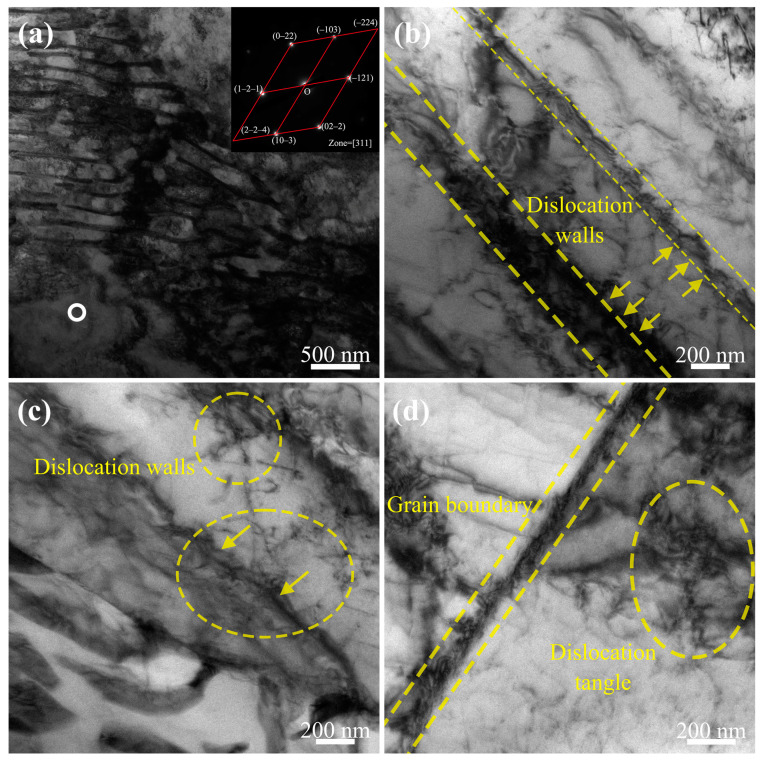
TEM micrograph of specimen 2#: (**a**) dislocation morphology of the specimen and the corresponding SAED pattern of the white-ring precipitation phase; (**b**) more microscopic morphology of the dislocation wall; (**c**) traces of dis-location movement toward the dislocation wall; and (**d**) dislocation morphology at grain boundaries.

The TEM micrographs and selected area electron diffraction (SAED) patterns for sample 2# (subjected to 20 cycles of ice plugging) are presented in Figure 12. As shown in Figure 12a, the dislocation density within the grains notably increases compared to samples 0# and 1#, with some grains being densely populated by dislocations. The SAED pattern indicates that sample 2# still consists of a ferrite phase with a body-centered cubic structure, suggesting that the phase composition does not change with an increase in the number of cycles. In Figure 12b,c, it can be observed that, due to the increased cycle count, the dislocation structure in sample 2# transforms from individual dislocation lines and low-density dislocation tangles into high-density dislocation walls. Furthermore, as seen in Figure 12c, there is a tendency for dislocations between two walls to migrate towards the walls, thereby thickening them. Additionally, it is evident from Figure 12c that low-density dislocation walls and tangles near grain boundaries tend to grow towards these boundaries. Consequently, the defining characteristic of the dislocation structure in sample 2# is predominantly high-density dislocation walls. Overall, as the cyclic strain accumulates, the interaction among dislocations progressively intensifies, leading to a more complex mechanism of dislocation–dislocation interactions [16,17].

**Figure 13:**
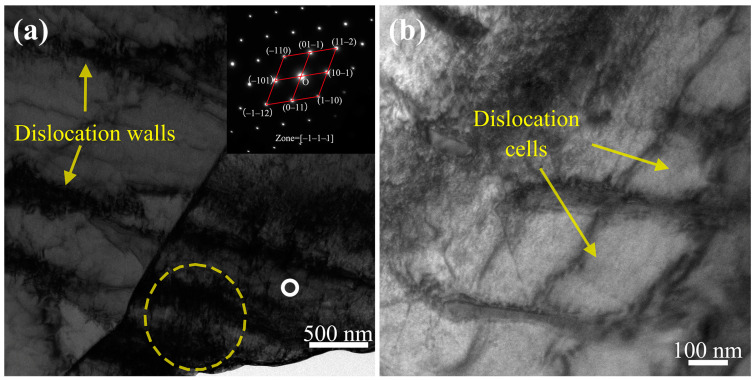
TEM micrograph of specimen 3#: (**a**) dislocation wall morphology of the specimen and the corresponding SAED pattern of the white ring precipitation phase; (**b**) dislocation cell morphology.

The TEM micrographs and selected area electron diffraction (SAED) patterns for sample 3# (having undergone 40 cycles of ice plugging) are depicted in Figure 13. As illustrated in Figure 13a, with the increment in the number of ice plugging cycles to 40, the cumulative plastic deformation continues to increase, consequently leading to a further escalation in the dislocation density. Moreover, Figure 13b shows that the buildup of plastic deformation results in more connections between dislocation walls, with some regions transforming into dislocation cells. Dislocation cells are characterized by high-density dislocation walls separated by areas of a lower dislocation density. At this stage, the dislocation structure predominantly features high-density dislocation walls and dislocation cells, interlaced with sparse dislocations.

Following the cyclic ice plug test, the strength (Figure 5) and hardness (Figure 6 and Figure 7) of the specimens exhibit an increase, while the plastic toughness experiences a decrease (Figure 6 and Figure 8). This strength improvement can primarily be attributed to the heightened dislocation density that arises within the samples after deformation [41,42]. The formation of ice plugs leads to an increase in the compressive force exerted on the tube walls due to the freezing of the liquid medium (water) (Figure 4), resulting in minor plastic deformation. Consequently, multiple cross-slip events occur during this plastic deformation, giving rise to a significant number of dislocations (Figure 11). Notably, cyclic plastic deformation leads to a substantial increase in dislocation density within the specimens (Figure 10b). Moreover, plastic deformation facilitates the movement, accumulation, and entanglement of existing dislocations, thereby increasing the internal rheological stresses in the material [43,44]. As deformation progresses, the dislocation density continues to rise, leading to high-density dislocation walls (Figure 12b,c) and dislocation cells (Figure 13b). These high-density dislocation walls and cells impede dislocation mobility, triggering the phenomenon of strain hardening [20,30]. Consequently, the yield strength, tensile strength, and microhardness of the specimens increase following cyclic ice plugging. However, accumulating many dislocations at the grain boundaries and increased dislocation density hinder further dislocation motions during plastic deformation [11,16,35]. In addition, the accumulation of dislocations during cyclic ice plug deformation gives rise to a significant stress field surrounding the dislocation buildup [45]. This stress concentration region becomes prone to crack formation, thereby increasing its susceptibility to brittle damage. Consequently, the plastic deformability of the specimen decreases following cyclic ice plugging, as evidenced by a reduction in the impact absorption work.

The investigation mentioned above of the mechanism underlying the impact of ice plug technology on nuclear-grade mild steel pipes offers valuable insights for developing a more rational piping design program, thereby enhancing the safety and reliability of the system. To mitigate the adverse effects of ice plug cycling on pipe materials, it is advisable to consider materials with a higher resistance to strain hardening during the selection and installation of piping. Furthermore, a comprehensive understanding of how ice plug cycling affects the plastic deformation and mechanical properties of pipeline materials can facilitate the development of more effective strategies for pipeline maintenance. Regular inspections and assessments of dislocation morphology and mechanical properties enable the timely detection of potential issues, facilitating the implementation of appropriate maintenance measures to ensure the secure operation of pipeline systems. Additionally, this study holds significance beyond the realm of nuclear-grade mild steel pipes as it provides a valuable reference for the application of ice plug technology in other fields. For instance, in the domain of industrial pipeline maintenance or other contexts necessitating the isolation of liquid-medium pipelines, leveraging the principles and safety performance of ice plug technology can contribute to the refinement of design and maintenance methodologies in related systems.

## 4. Conclusions

Cyclic ice plugging tests were conducted on nuclear-grade 20# pipeline steel to examine the dislocation morphology and mechanical properties of the specimens following cyclic ice plugging. Based on the analysis of the experimental results, the following conclusions can be drawn:

(1) As ice plugs gradually formed within the pipe, the cyclic stress on the pipe wall increased steadily, reaching −46 MPa. This indicates an intensified compression effect of the ice plugs on the pipe wall. After numerous ice plugging cycles, the pipeline material’s uncircularity increases, accompanied by a slight plastic deformation. As a result, the safety of the pipeline for sustained service in the nuclear power system is somewhat compromised.
(2) The mechanical properties of the pipe material changed as a result of cyclic ice plugging. The strength and hardness increased with the number of ice plug cycles. For instance, after 40 cycles of ice plugging, the yield strength and tensile strength of the specimens increased from 285.56 MPa and 452.71 MPa, respectively, in the original specimens to 301.15 MPa and 477.48 MPa. Similarly, the microhardness of the specimens increased by approximately 19.43%. Conversely, the impact toughness of the specimens displayed alterations, exhibiting a gradual decrease in toughness fracture characteristics. This decrease in impact toughness reduces the safety of pipeline usage. Hence, it is advisable to avoid multiple ice plugging operations at the same location of the pipe when employing ice plugging for maintenance purposes.
(3) The accumulation of microplastic deformation induces the proliferation and mobility of dislocations, exacerbating dislocation interactions such as stacking and entanglement. Following three cycles of ice plugging, there is an increase in the number of dislocations within a specimen’s internal structure, with the dislocation density rising from 5.43 × 10^13^ m^−2^ to 7.34 × 10^13^ m^−2^. The prevailing dislocation morphology is characterized by a low-density dislocation entanglement. As the number of ice plugging cycles reaches 20, the dislocation density within the specimen escalates to 1.18 × 10^14^ m^−2^, accompanied by a shift in the micro-dislocation morphology towards high-density dislocation walls. Upon reaching 40 ice plugging cycles, the dislocation density further rises to 1.42 × 10^14^ m^−2^, and the dislocation pattern transitions to dislocation cells, characterized by a lower dislocation density within the cells.
(4) The presence of high-density dislocation walls and dislocation cells restricts the mobility of dislocations, resulting in an enhancement of the strength and hardness of the pipe wall material. The elevated dislocation density impedes the continued movement of dislocations during plastic deformation, consequently reducing the impact toughness of the pipe material. These alterations in the mechanical properties of pipeline materials following ice plug cycling offer a practical reference for the selection of nuclear power pipelines and the development of more effective pipeline maintenance strategies.

## Figures and Tables

**Table 1:** Chemical composition of the experimental steels (wt %).

C	Si	Mn	P	S	N	Cr	Cu	Ni	Fe
0.187	0.309	0.796	<0.005	0.043	0.0674	0.0387	0.122	0.0511	Bal.

**Table 2:** The tensile properties of 0#, 1#, 2#, and 3#.

Sample	Yield Strength (MPa)	Tensile Strength (MPa)	Total Elongation (%)
0#	285.56	452.71	42.35
1#	286.12	459.77	42.01
2#	299.38	470.92	40.19
3#	301.15	477.48	39.20

## Data Availability

Data are contained within the article.

## Abbreviations

HWR	Heavy-Water Reactor
TEM	Transmission Electron Microscopy
HV	Vickers’ Hardness
SEM	Scanning Electron Microscopy
XRD	X-ray Diffractometer
BCC	Body-Centered Cubic
MWH	Modified Williamson–Hall
SAED	Selected Area Electron Diffraction
